# The burden of nosocomial superinfections in a retrospective cohort study of critically ill COVID-19 patients

**DOI:** 10.1186/s12879-025-10983-7

**Published:** 2025-05-03

**Authors:** Anne Kathrin Lösslein, Paulina Ines Staus, Cynthia Beisert Carneiro, Martin Wolkewitz, Georg Häcker

**Affiliations:** 1 Institute of Medical Microbiology and Hygiene, Medical Center and Faculty of Medicine, University of Freiburg, Freiburg, Germany; 2 Institute for Immunodeficiency, Center for Chronic Immunodeficiency, Medical Center and Faculty of Medicine, University of Freiburg, Freiburg, Germany; 3 Institute of Medical Biometry and Statistics, Division Methods in Clinical Epidemiology, Medical Center, Faculty of Medicine, University of Freiburg, Freiburg, Germany

**Keywords:** Intensive care unit, COVID-19, Healthcare associated infections, Superinfections, Competing risks, Hospital mortality, Length of stay

## Abstract

**Objectives:**

Viral respiratory infections can be complicated by bacterial superinfections. SARS-CoV-2 patients may suffer from superinfections, and negative effects of additional infections have been identified. When analysing hospital data, patients typically leave the facility of observation, due to discharge or death, which leads to changes in the study cohort over time. This may distort the estimate of the impact of superinfection. Therefore, it is essential for the statistical analysis of hospital data to acknowledge this change of the risk set over time. We analysed superinfections in a retrospective cohort study with 268 critically ill patients, taking into account discharge and death as competing risks in the statistical analysis.

**Methods:**

We evaluated bacterial respiratory infections and bloodstream infections and used multi-state statistical modelling to account for the different patient states. We calculated risks of superinfection, probability of discharge or death over time and analysed subgroups according to age and sex.

**Results:**

The observed pathogen spectrum was mainly composed of *Enterobacterales*, *Nonfermenters* but also *Staphylococcus aureus*. We identified an elevated mortality due to bacterial infection of the respiratory tract or bloodstream infection (adj. cause-specific HR 1.7, CI 1.15–2.52) as well as a reduced discharge rate (adj. cause-specific HR 0.51, CI 0.36–0.73). Female patients showed a tendency to have a reduced risk of acquiring a superinfection (adj. subdistribution HR 0.71, CI 0.48–1.04), and in case of infection a higher mortality compared to male patients (interaction effect HR 1.49, CI 0.67–3.30).

**Conclusions:**

The study accounts for competing risks and quantifies the risk of death associated with bacterial superinfection in critically ill COVID-19 patients. We observed an increased risk of death for patients who developed a superinfection, with *Enterobacterales* being the predominant agent. The results emphasize the need for microbiological sampling in SARS-CoV-2-infected patients.

**Clinical trial number:**

German Clinical Trials Register number: DRKS00031367, registration date: 01.03.2023 (https://drks.de/search/de/trial/DRKS00031367).

**Supplementary Information:**

The online version contains supplementary material available at 10.1186/s12879-025-10983-7.

## Introduction

Infections with respiratory viruses, best studied for influenza virus, carry the risk of superinfections by bacteria and fungi. Such superinfections can critically add to the burden of disease and increase lethality (reviewed in [1, 2]). It is plausible that infections with SARS-CoV-2, with a wide spectrum of clinical presentation and outcome, may also be associated with superinfections, and therefore a number of studies have investigated the occurrence and outcome of superinfections in COVID-19-patients (e.g. [3–5]). Most studies find a relatively low rate of bacterial co-infections at first presentation with SARS-CoV-2 but a higher rate of secondary bacterial infections in hospitalized patients, i.e. infections acquired during the course of COVID-19 within a clinical facility [6, 7]. Assessing risk and risk factors for hospital acquired infections is challenging because some patients leave the hospital due to discharge or death before an infection can occur. Many studies estimating superinfection rates using hospital data do not account for these events (e.g. [7]), which can lead to biased results. Often, patients who leave the hospital are simply considered ‘censored’ in the analysis, but this approach has been shown to significantly distort risk estimates [8]. Accordingly, it is not precisely known at this stage how a superinfection changes the risk of death or the likelihood of discharge from hospital, how patient characteristics such as sex and age affect these risks, or how these risks evolve over time during COVID-19 treatment in the inpatient setting.

We addressed these questions using multi-state models and cause-specific Cox models. Our analysis is based on a cohort of COVID-19 patients hospitalized at a tertiary care hospital in the South of Germany. For the statistical analysis, we focussed on superinfections (respiratory infections and bloodstream infections) that patients acquired during treatment at intensive care units (ICUs). We implemented superinfection as a time-varying variable in the statistical analysis to take the timing of a superinfection into account when estimating its influence on the risk of death and discharge in COVID-19 patients. We further assessed how a superinfection affects the duration of hospitalization in age- and sex-specific subgroups of our cohort.

## Methods

### Study design and patient cohort

For this retrospective study cohort, we included adult patients who received treatment for COVID-19 from February 2020 to 20^th^ July 2021 at the University Medical Center Freiburg and were admitted to the ICU during their hospital stay. Patients who tested positive for SARS-CoV-2 were recorded by the Division of Infectious Diseases of the Medical Center Freiburg. We excluded children, pregnant women who were in hospital for delivery without COVID-19-associated complications and patients who presented in the emergency department but were not admitted to stationary care. In addition, we excluded patients in whom COVID-19 infection was asymptomatic or otherwise was not a cause of admission and did not affect the course of their stay. This was individually evaluated for each patient from the patient records. The second admissions of 3 patients were excluded. We recorded sex, age, outcome, length of stay and periods in intensive care units for these patients (short-term stays for invasive procedures such as punctures were not considered). Superinfections were determined as described below. Patients with nosocomially acquired SARS-CoV-2 infections (*n* = 60; positive PCR at the earliest 3 days after the start of hospitalization) and superinfections on the first day of their hospital stay were also excluded. Of the remaining patients, only individuals who were admitted to the ICU during their hospital stay were included in the statistical analysis, and we only considered their first ICU stay. Finally, patients with an event (superinfection, discharge or death) at the day of ICU admission were excluded from the statistical analysis (Supplementary Fig. 1).

### Microbiological analysis

We performed a retrospective data query (in 2020 and 2021) in the laboratory software [M/lab] (DORNER Health IT Solutions) for the routine microbiological results obtained during the patients’ hospital stay. Clinical information regarding the patients (including antibiotic treatment and patient records) was extracted from the clinical information system MeDoc. For the statistical analysis, a study number was assigned for pseudonymization.

We considered *Candida* in blood cultures and pathogenic bacteria, both in respiratory materials and in blood cultures. Superinfections with filamentous fungi and viruses were not considered in this analysis, because the data set was not comprehensive for PCR and serology diagnostics required to detect these agents. For the evaluation of the relevance of the microbiological results, we defined the following criteria and considered each microbiological result individually: common blood culture contaminants (e.g. skin commensals and environmental microbes) were disregarded if they were only detected in one sample. In case of doubt, we considered whether antibiotic therapy had been administered for the infectious agent detected. Respiratory samples included bronchoalveolar lavage, bronchial and tracheal secretion and sputum. For respiratory materials, we used the following three criteria to define relevance of the isolated pathogen: first, the pathogen species was isolated from more than one respiratory sample; secondly, the sample was of high quality (presence of neutrophils, indicative of inflammation, and no or little contamination by squamous epithelium); thirdly, the isolated agent was considered clinically relevant in the respective patient (based on the clinical patient record). If 2 of the 3 criteria were met, we regarded the pathogen as relevant. *Candida*, *Enterococci* and coagulase-negative *Staphylococci* were considered relevant only when isolated from blood culture. When the same species of pathogen was found in both respiratory material and blood culture, they were always considered relevant. In six patient cases, clinical records or the microbiological sampling were insufficient to decide with the defined criteria on the presence of a respiratory superinfection, so that the isolated pathogens were disregarded. While patients may acquire several superinfections during the hospital stay, we considered only the first superinfection for the statistical analysis.

### Outcomes

Of primary interest is the burden of nosocomial superinfection for COVID-19 patients within an ICU unit. In detail, we estimated the increase in mortality associated with superinfection and the extra days a patients stays longer/shorter within the ICU on average associated with superinfection. Mortality estimates are based on hospital death within 90 days after ICU admission. We highlight their correct estimation, taking into account the change of the risk set for superinfection over time due to the competing outcomes discharge and death. Of secondary interest were the description of pathogens causing superinfections in COVID-19 patients in our setting and the influence of sex and age on acquiring superinfections. For a complete picture of the process, we investigated the influence of sex and age on the risk of discharge and death, in addition. Subgroup analysis of age and sex were prespecified and the only subgroup analysis investigated.

### Statistical analysis

As patients who have been discharged from the hospital are no longer at risk for the primary endpoint, simple Kaplan–Meier estimates are biased [9, 10]. Instead, we fitted a multi-state model with the states ICU admission (0), superinfection (1), hospital discharge without superinfection (2), in hospital death without infection (3), hospital discharge with superinfection (4), in hospital death with superinfection (5), with the transitions 01, 02, 03, 14, 15, and superinfection meaning detection of the infection according to the criteria above at any time point after the first ICU day (Supplementary Fig. 2). For the statistical analysis, only the first superinfection event was considered. We performed all analyses in R version 4.2.2 (https://www.R-project.org/). We used the mvna package (2.0.1) [11] to derive the cumulative hazard function with the Nelson Aalen estimator, the alternative for the Kaplan–Meier estimator in a competing risk setting, and used the etm package (1.1.1) [12] to derive the transition probabilities between the states (see result section patient hospital outcomes). If patients had two events on one day (e.g. superinfection and death/discharge) a small time interval of 0.0001 days was added to satisfy model assumptions. The day of ICU/hospital admission was coded as day 1.

We calculated cause-specific Cox-models using the survival package (3.5–5) [13] to derive the HR of superinfection for the endpoint hospital death and the competing endpoint hospital discharge. The Cox-models were adjusted for age and sex as common confounders. These variables were selected due to expert knowledge from the limited available information. We assessed the proportional hazard assumption visually looking at cumulative hazard curves.

We performed a proportional subdistribution hazard regression using the cmprsk package (2.2–11) [9] to evaluate the influence of age and sex on the risk of acquiring a superinfection, of discharge without a superinfection, and of death without superinfection. Thereby, we were able to distinguish if age and/or sex is associated with an intrinsic property to be more susceptible to acquire a superinfection, or if age and sex were associated with the duration under risk to acquire a superinfection.

To evaluate the burden of superinfections for resource allocation we calculated the number of mean days a patient stays longer in the hospital associated with the superinfection. To account for the time of acquiring a superinfection we fitted a multi-state model with the states admission to ICU (0), superinfection (1), end of stay (2) with the transitions 01, 12, 02 (Supplementary Fig. 3). We calculated the mean additional length of stay using the clos function of the etm package. 95% confidence intervals (CI) for extra length of stay were calculated by bootstrapping 100 times.

## Results

### Demographic data of the patient cohort

In the period under consideration, 281 adult COVID-19 patients received treatment at an ICU at the University Medical Center Freiburg, a tertiary care hospital with approximately 1,600 beds in Southern Germany. As outlined in detail in the methods, this already excludes patients with nosocomial SARS-CoV-2 infections and superinfections on day 1 of their hospital stay. To circumvent selection bias, patients who had already acquired a superinfection, were discharged or died on the first day of ICU stay were also excluded (any infection *n* = 6, discharge = 1, death = 6, Supplementary Fig. 1). The final cohort consisted of 268 SARS-CoV-2 patients admitted to the ICU, event free at the day of ICU admission (Supplementary Fig. 1). The statistical analysis was performed on these 268 patients. Of these patients, 194 (72%) were male, and the median age was 60 years (Table 1). In our patient cohort, 144 acquired a respiratory superinfection and/or bloodstream infection (‘any superinfection’), (53.7%). All but three patients of the cohort acquired them during their first ICU stay. Bloodstream infections were considered separately and, constitute a subgroup of superinfections, (Table 1). The composition of the ICU patient cohort is shown in Table 1.

**Table 1:** Baseline characteristics of the patients in the cohort under investigation

Variables	Total	Superinfection		Bloodstream infection	
		No	Yes	No	Yes
	*N* = 268	*N* = 124	*N* = 144	*N* = 198	*N* = 70
Sex					
Male	194 (72%)	83 (67%)	111 (77%)	140 (71%)	54 (77%)
Female	74 (28%)	41 (33%)	33 (23%)	58 (29%)	16 (23%)
Age (years)					
Mean (SD)	60 (± 10)	60 (± 20)	60 (± 10)	60 (± 10)	60 (± 9)
Median (Q1, Q3)	60 (50, 70)	60 (50, 70)	60 (50, 70)	60 (50, 70)	60 (60, 70)
Hospital stay (days)					
Mean (SD)	30 (± 20)	20 (± 20)	30 (± 20)	20 (± 20)	30 (± 20)
Median (Q1, Q3)	20 (10, 30)	20 (8, 20)	30 (20, 40)	20 (10, 30)	30 (20, 50)
ICU stay (days)					
Mean (SD)	20 (± 20)	10 (± 10)	30 (± 20)	20 (± 20)	30 (± 20)
Median (Q1, Q3)	20 (7, 30)	8 (4, 20)	20 (20, 40)	10 (6, 20)	20 (20, 40)
Status					
Alive	145 (54%)	77 (62%)	68 (47%)	120 (61%)	25 (36%)
Dead	123 (46%)	47 (38%)	76 (53%)	78 (39%)	45 (64%)

Baseline characteristics (age, sex, length of ICU stay and status at discharge) of the included ICU patients (*n* = 268) in the cohort under investigation

### The pathogen spectrum detected in the COVID-19 patient cohort

**Fig. 1:**
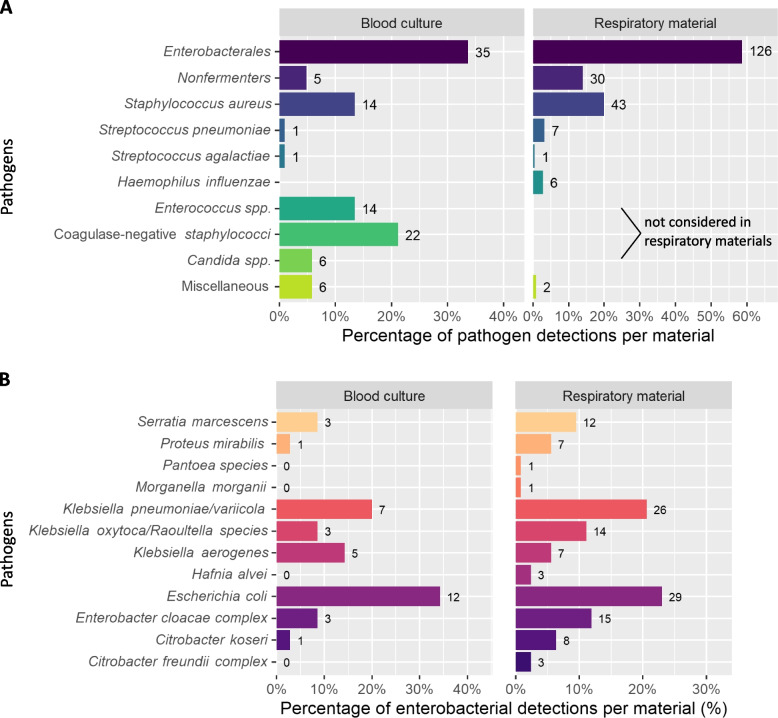
Spectrum of pathogens detected in the COVID-19 patient cohort. **A** Spectrum and number of relevant pathogens detected either in blood cultures (left) or in respiratory material (right) during the whole hospital stay. The length of the bars gives the percentages of all detected pathogens in the respective material. **B** Breakdown of *Enterobacterales* from (A) by species. The length of the bars gives the percentages of all detected *Enterobacterales* in the respective material

In our cohort, blood culture sampling was performed in the majority of patients (94.8%) whereas respiratory materials were obtained in 83.6% of the 268 patients. Figure 1A shows the frequencies and absolute numbers of all pathogens that were detected during the hospital stay in association with a superinfection. In this graph, we considered not only the first episode of a superinfection, so that an individual patient may be listed more than once (Fig. 1A, Supplementary Table 1). In addition, Supplementary Fig. 4 depicts the number and percentages of patients with at least one of the pathogens detected in blood culture or respiratory material (Supplementary Fig. 4). The most frequent pathogens were *Enterobacterales* (126 detections in respiratory materials [58.6% of detected pathogens] in 96 patients [35.82% of all patients] and 35 detections in blood culture [33.65% of detected pathogens] in 32 patients [11.94% of all patients]) with a predominance of *Escherichia coli* and *Klebsiella species,* in both bloodstream and respiratory infections (Fig. 1A and B, Supplementary Fig. 4, Supplementary Tables 1 and 2), followed by *Staphylococcus aureus* at both sites (43 detections in respiratory materials [20% of detected pathogens] in 43 patients [16.04% of all patients] and 14 detections in blood culture [13.46% of detected pathogens] in 14 patients [5.22% of all patients]). Non-fermenting bacteria were also isolated at relatively high frequencies from both types of material (30 detections in respiratory materials [13.95% of all detected pathogens] in 29 patients [10.82% of all patients] and 5 detections in blood culture [4.81% of all detected pathogens] in 5 patients [1.87% of all patients]), (Fig. 1A and B, Supplementary Fig. 4, Supplementary Tables 1 and 2). By blood culture, coagulase-negative *Staphylococci*, *Enterococci* and *Candida* were also isolated; these agents were considered non-pathogenic in the respiratory tract and therefore ignored in materials from that site. In 34 of 268 patients (12.69%), we identified the same pathogen in blood culture and respiratory material with identical antibiotic susceptibility.

### Patient hospital outcomes

In our cohort the estimated probability to acquire any superinfection within the first 90 days after ICU admission within the hospital was 53.7% (CI 47.8–59.7, *n* = 144). The probability of hospital death within 90 days after ICU admission without acquiring a superinfection before was 19% (CI 14.3–23.7, *n* = 51). Death considered here is either death within the ICU or the subsequent stay on the normal ward. The remaining patients were discharged (27.2%, *n* = 73) and no further information on living status was available. Of 144 patients who acquired a superinfection within the hospital 78 died within the first 90 days after ICU admission, 63 were discharged and 3 were still cared for within the hospital. Patients with a superinfection from the start of ICU stay have a probability to die within the hospital within 90 days of 61% (CI 52.6–69.3), a probability to be discharge within 90 days of 37.6% (CI 29.3–45.8) and a probability to still being cared for within the hospital within 90 days of 1.4% (CI 0–3.1).

### Influence of superinfections on the clinical outcome

Superinfections (both any superinfection and bloodstream infection) were associated with an increased risk of death and reduced likelihood of discharge. After adjusting for age and sex, patients had a 1.7 fold (adj. cause-specific hazard ratio (HR), confidence interval (CI) 1.15–2.52) increased mortality rate if acquiring any superinfection and a 2.7 fold (adj. cause-specific HR, CI 1.8—3.9) increased mortality rate if acquiring a bloodstream infection (Table 2A and B). Superinfection and bloodstream infection reduced the hazard to be discharged. Patients’ discharge rate was reduced by 49% (CI 27%–64% adj. cause-specific HR), and 57% (CI 31%–73%, adj. cause-specific HR) respectively. Increased age was associated with mortality independently of superinfection. In addition, in the analysis of bloodstream infections, each year of age was associated with a reduction of the discharge rate by 1% (CI 21%− 0%, *p* = 0.040, cause-specific HR, Table 2).

**Table 2:** The influence of superinfections on the hazard for hospital discharge or hospital death

**A Any Superinfection**								
Outcome	Hospital Discharge				Hospital Death			
Predictors	HR	95% CI	*p*-value		HR		95% CI	*p*-value
Superinfection	0.51	0.36; 0.73	** < 0.001**		1.70		1.15; 2.52	**0.008**
Age (years)	0.99	0.97; 1.00	0.074		1.03		1.02; 1.05	**< 0.001**
Sex [female]	1.15	0.79; 1.68	0.463		1.14		0.77; 1.68	0.523
R^2^ Nagelkerke	0.048				0.058			
**B Bloodstream infection**								
Outcome	Hospital Discharge				Hospital Death			
Predictors	HR	95% CI	*p*-value		HR		95% CI	*p*-value
Superinfection	0.43	0.27; 0.69	**0.001**		2.66		1.80; 3.93	**﻿< 0.001**
Age (years)	0.99	0.79; 1.00	**0.040**		1.04		1.02; 1.05	**< 0.001**
Sex [female]	1.24	0.85; 1.80	0.262		1.11		0.75; 1.64	0.594
R^2^ Nagelkerke	0.057				0.110			

The influence of (A) any Superinfection and (B) bloodstream infection on the hazard for hospital discharge (left) or hospital death (right), estimated by cause-specific Cox regression with time-varying superinfection status, adjusting for age and sex (*n* = 268). We implemented age as a continuous variable using years as unit. *CI* Confidence intervals, R2 Nagelkerge: coefficient of determination which quantifies how much variation of the data is explained by the model

### Transition probability: time course and mortality of superinfections

**Fig. 2:**
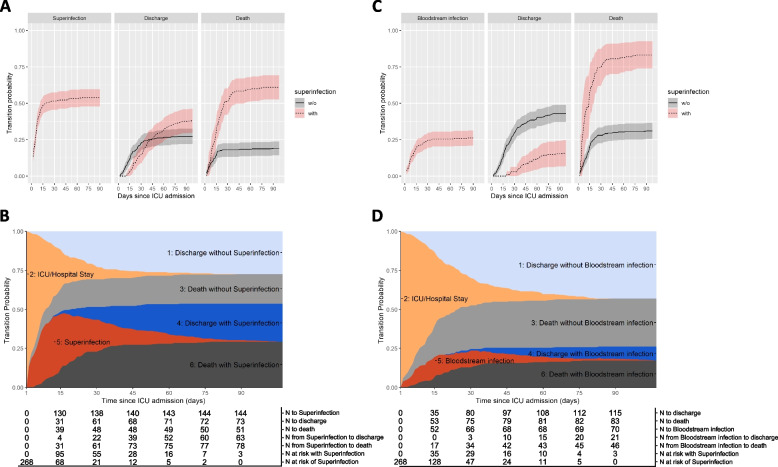
Mortality associated with superinfections. **A** Transition probability: any superinfection. **B** Stacked probability plot: any superinfection. **C** Transition probability: bloodstream infection. **D** Stacked probability plot: bloodstream infection

The hazard rate of acquiring the first superinfection was highest at the start of ICU stay and decreased continuously over time (Fig. 2A, Supplementary Fig. 5). Superinfection delayed the probability to be discharged and the risk of hospital death was greater for patients who acquired a superinfection (Fig. 2A).

A comprehensive way of visualizing the outcome for the whole cohort is the presentation as ‘stacked probability plots’ (Fig. 2B). Patients arrive at the ICU without superinfection (light orange area) and may acquire a superinfection (orange-red). They may be discharged without superinfection (light blue) or following acquisition of a superinfection (dark blue), or may die without (light grey) or with acquisition (dark grey) of a superinfection (Fig. 2B). In the absence of superinfection, more than half of the patients were discharged, while over half of the patients with superinfection died (Fig. 2B, comparing proportions of blue against grey at the end of follow-up). Principally similar but even more striking results were obtained when we analysed only the bloodstream infections (Fig. 2C, D). In the absence of bloodstream infection, probability of death was lower than probability of discharge, while the probability of death was about twice as high as probability of discharge when patients had acquired a bloodstream infection (Fig. 2D).

### Risk factors for superinfections

**Fig. 3:**
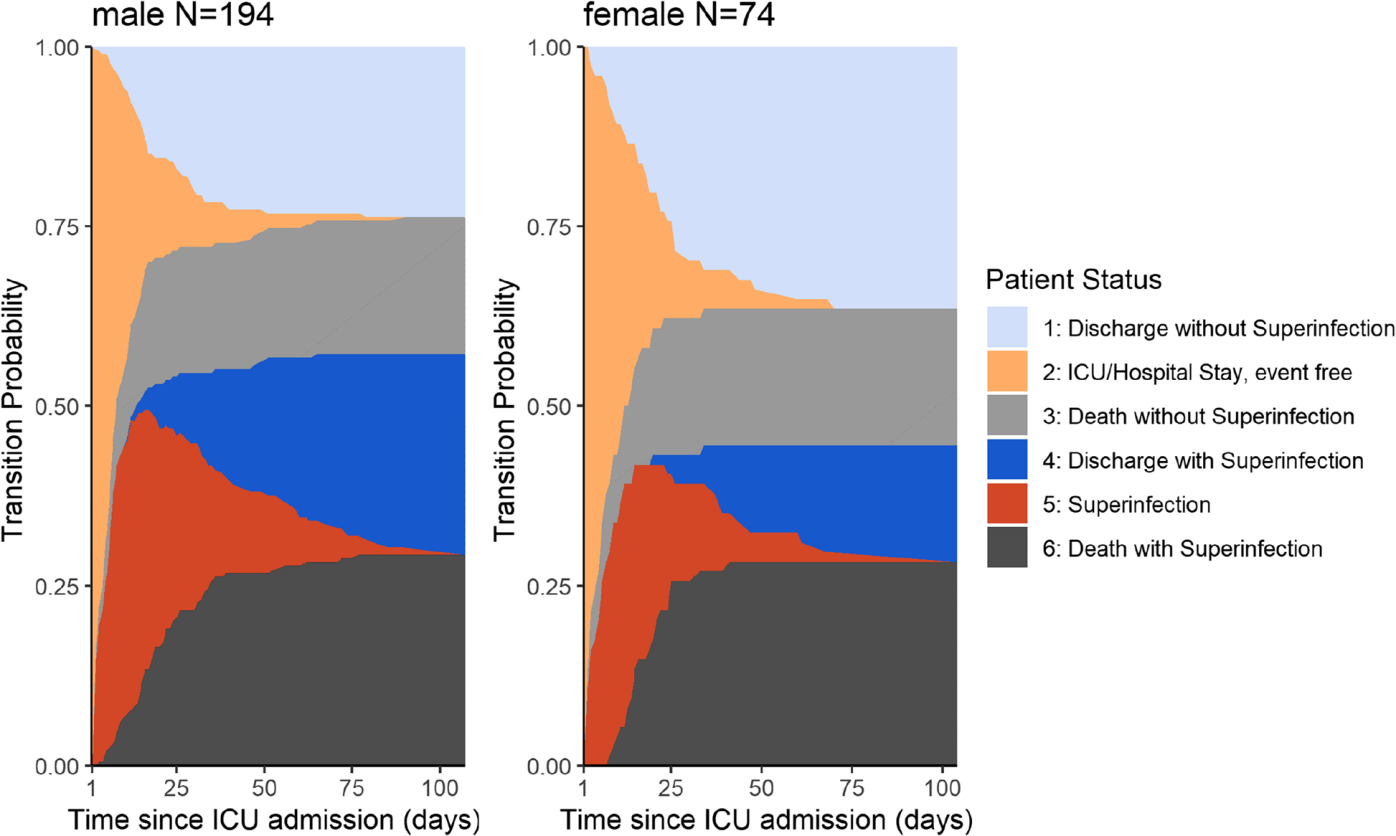
Transition probability: subgroup analysis stratifying for sex

To identify patient risk factors for superinfection and outcome in COVID-19 infection, we analysed the risk of superinfection, discharge and death according to sex and age. Female patients had a reduced hazard of superinfection (compare the proportions of dark blue and grey to light blue and grey, Fig. 3; cause-specific hazard ratio (HR): 0.73 95% CI 0.49–1.08, *p*-value 0.112). Female patients acquired superinfections less frequently (light blue; subdistribution hazard ratio 0.71 95% CI 0.48–1.04, *p*-value 0.080). Those who did, tended to have a higher probability of death in comparison to male patients (comparison of dark grey with dark blue, Fig. 3, interaction effect non-significant HR 1.49 95% CI 0.67–3.30, *p*-value 0.324).

**Fig. 4:**
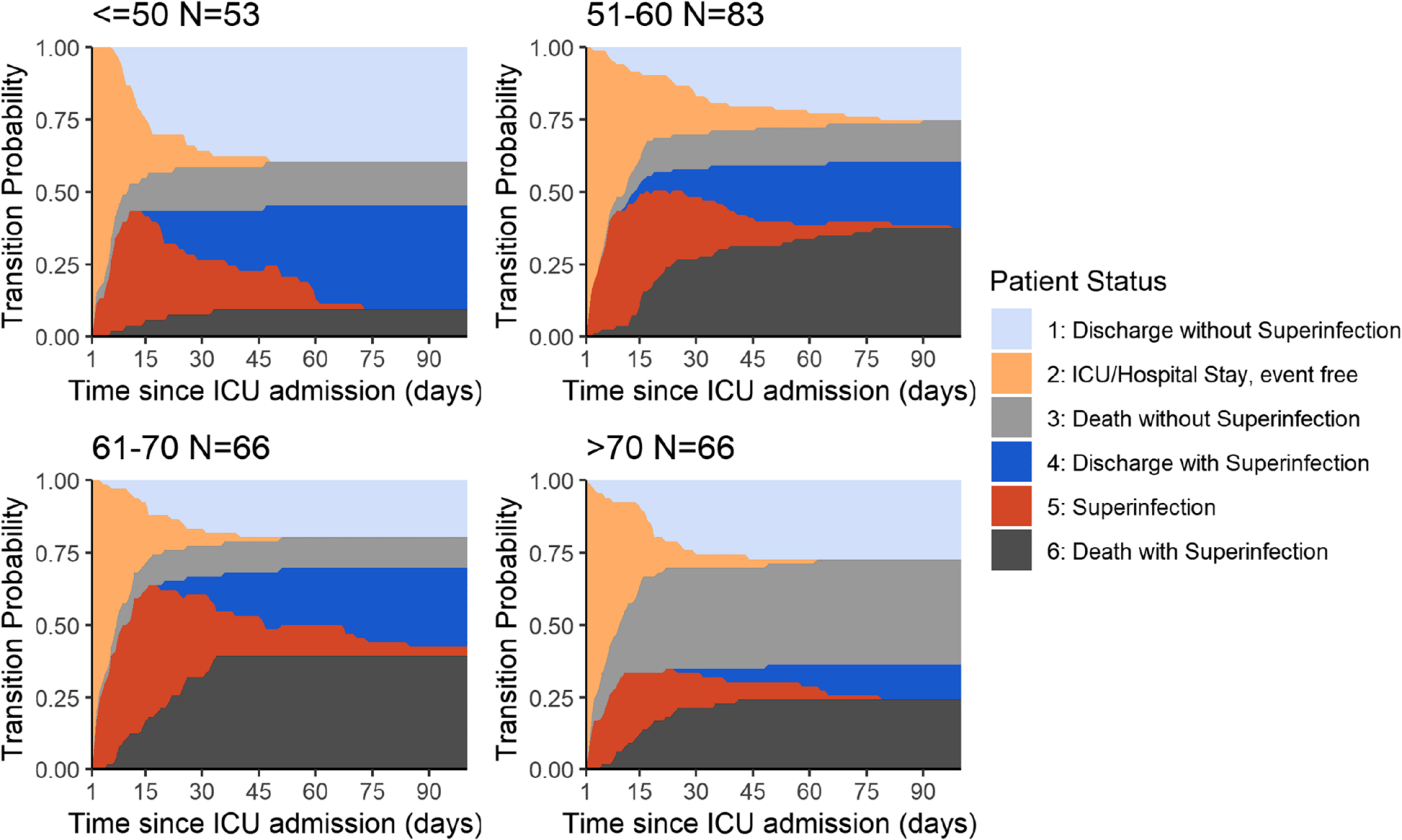
Transition probability: subgroup analysis stratifying for age

Younger COVID-19 patients survived superinfections to a higher percentage than older patients (compare dark blue compared to dark grey in the four age groups, Fig. 4). With age, patients tended to acquire superinfections with higher probability (fraction of dark blue and dark grey together compared to light blue and light grey at the end of the time period). With increasing age, patients spent more time on the ICU before being discharged, associated with an increased time at risk of acquiring a superinfection. Very old patients > 70 years acquired proportionally fewer superinfections than younger patients, as shown by the smaller combined dark blue and grey area, (Fig. 4). This is primarily due to a higher mortality rate in the older age group (represented by the large light grey area) preventing them to develop a superinfection. The rate for death without infection increases by 4% for each year of age (1.04 cause-specific HR for death without infection, 95% CI: 1.02–1.07, *p*-value: 0.001) (Table 2).

### Extra length of stay

We calculated the effect of acquiring a superinfection on the length of stay on the ICU, considering the combination of the effects of superinfection on discharge and on death. COVID-19 patients did not stay significantly longer in hospital due to superinfection (Table 3). Only in the subgroup of patients younger than 51 years of age, a significantly prolonged stay in hospital was observed (average 9 extra days, 95% CI 0.07–17.03), (Fig. 4). When looking at the effect of bloodstream infections (*n* = 70) on the length of stay, the estimates are less precise. Although not significant, the first two age groups show a prolonged ICU stay in the point estimates associated with bloodstream infections, whereas similar to the effect of any superinfection 61 to 70 year old patients showed a reduced ICU stay point estimate.

**Table 3:** Superinfections and length of stay

**A** Any Superinfection											
Group	Extra length of stay in ICU					Median days in hospital* after ICU discharge with infection			Median days in hospital* after ICU discharge without infection		
	Days (95% CI)			*n*		Days (IQR)	*n*		Days (IQR)		*n*
Total	− 1.28 (- 5.82; 3.34)			268		0.0 (0; 7)	65		4.0 (0; 9)		80
Age											
< = 50	9.00 (0.07; 17.03)			53		2.5 (0; 7.75)	18		6.0 (4; 7.5)		23
51–60	− 7.13 (− 15.81; 1.40)			83		0.0 (0; 6.5)	19		3.0 (0; 5)		22
61–70	− 5.16 (− 19.73; 7.64)			66		0.0 (0; 2.75)	20		4.0 (0; 8)		13
> 70	2.04 (− 8.89; 13.09)			66		0.5 (0; 4.75)	8		7.5 (2; 15)		22
Sex											
Male	− 2.92 (- 7.12; 2.71)			194		0.0 (0; 7.75)	54		4.0 (0; 7.75)		50
Female	0.61 (- 9.06; 9.54)			74		0.0 (0; 0)	11		7.0 (3; 14.75)		30
**B** Bloodstream infection											
Group	Extra length of stay in ICU					Median days in hospital* after ICU discharge with infection			Median days in hospital* after ICU discharge without infection		
	Days (95% CI)			*n*		Days (IQR)	*n*		Days (IQR)		*n*
Total	0.97 (− 3.47; 6.07)			268		0.0 (0; 0)	21		4.0 (0; 9)		124
Age											
< = 50	3.25 (− 6.33; 11.44)			53		8.0 (0; 15)	5		4.5 (0; 7)		36
51–60	2.60 (− 5.96; 14.31)			83		0.0 (0; 0)	6		3.0 (0; 6.5)		35
61–70	− 5.11 (− 12.1; 6.74)			66		0.0 (0; 0)	7		1.0 (0; 9.5)		26
> 70	1.55 (− 5.58; 17.26)			66		0.0 (0; 0)	3		7.0 (1; 15)		27
Sex											
Male	0.10 (− 6.19; 5.09)			194		0.0 (0; 0)	17		4.0 (0; 8)		87
Female	4.15 (− 7.89; 16.38)			74		0.0 (0; 7.25)	4		5.0 (0; 14)		37

Extra length of stay and median days in hospital after ICU discharge analysed dependent on age and sex for (A) any superinfection and (B) bloodstream infections. The extra length of stay is interpreted as estimated mean days an individual stays longer in the ICU associated with acquiring a superinfection, compared to a patient not acquiring a superinfection. (*Time from first ICU discharge to either hospital discharge, in hospital death or second ICU admission. 12 were readmitted to ICU, additional 2 patients died in hospital.)

## Discussion

Unfavourable effects of bacterial superinfections on the outcome of COVID-19 patients have been reported several times in the past [14, 15]. However, because patients leave the hospital and are no longer at risk of death within, a precise assessment of hospital mortality is only possible when considering this competing event, as we have done here. Our study identified a clear relevance of superinfections in COVID-19 patients requiring intensive care. Patients who acquired a superinfection had a substantially increased hazard of death, which varied according to age. These results emphasize the role of bacterial superinfections and the importance of identification and appropriate specific treatment of infected patients.

The spectrum of bacterial pathogens reported has been variable between studies [16–18], possibly indicative of different medication routinely used during treatment (such as prophylactic antibiotics and corticosteroids), different settings, and different infection prevention procedures, especially under the pressure of high numbers of COVID-19 patients. It is noteworthy that the different pathogen spectra detected in superinfections of COVID-19 patients might be associated with different SARS-CoV-2 infection waves and virus variants during the pandemic [19, 20]. In our cohort, the spectrum of microbial agent varied from *Enterobacterales* over *S. aureus* to *Nonfermenters*. A similar bacterial spectrum has also been described in other studies and is consistent with nosocomial infections [21]. The study by Schwaber et al. [22] also emphasized that nosocomial infections are particularly important for COVID-19 patients, while community-acquired infections seem to play a subordinate role [23].

Influenza patients are at particular risk of superinfection with *S. aureus* and *S. pneumoniae* [1]. While the cause of this strong association is unclear, it suggests a particular pathophysiological change in the lung of influenza patients, possibly associated with interferons [24], which may not be present in SARS-CoV-2-infected patients. We detected *Staphylococcus aureus* superinfection in 43 out of 268 ICU patients (16%) while *S. pneumoniae* was only rarely found. Additionally, we observed a high concordance of pathogen species isolated from respiratory material and from the blood in 34 of 268 patients (12.7%), suggestive of a high proportion of episodes of septic distributions of the bacteria from infected lungs.

In line with our observations, a multi-center study reported a high incidence of superinfection with *Enterobacterales* (64%) and *S. aureus* (28%) in VAP of COVID-19 patients at the ICU in Italy [25].

The core of our study is the multi-state modelling of superinfections in ICU patients, which allows us to analyse risks although the patient cohort is subject to constant change due to death and discharge and that the observed patient population shrinks over time [26]. We were able to show that most initial superinfections occurred during the first 20 days of admission to ICU. Considering the timing of superinfection when estimating extra length of stay due to superinfection is essential. When only data on hospital mortality are known, hospital discharge needs to be taken into account when estimating absolute risk and risk factors of in hospital death, as we did in the multi-state approach. A study using Kaplan–Meier estimates and other statistical methods reported an extended stay in a cohort of patients with a high rate of superinfections with multi-resistant bacteria [27, 28]. In our setting, applying the multi-state modelling approach, we could not confirm this effect for the ICU patient collective. Only for the patients younger than 51 years we calculated an extra length of stay of approximately nine days (CI 0.07- 17.03). One possible explanation for the negative values in extra length of stay associated with superinfection (Table 3) is the higher mortality rate in older age groups compared to those under 51. This results in a more pronounced ‘dying faster with superinfection’ effect, ultimately reducing the length of hospital stay in these patients (Fig. 4). Many patients with superinfections do not stay on a normal ward because they are directly transferred to external hospitals, specialised weaning centers, or rehabilitation clinics. These factors were not included in our data. We only considered the first ICU episode and first detected superinfection because there were only a limited number of re-ICU-admissions. This is a simplification of the transitions experienced by patients in the real world. Our models assume a constant effect of superinfection on mortality over time, which might average truly time-varying effects. The significant association of superinfection and increased mortality that we found using multi-state modelling was not observed by Tan et al. in a cohort of 136 COVID-19 patients receiving ECMO [21]. However, it must be taken into account that Tan et al. based their analysis on different statistical models [21]. As in all cases of infection, microbiological diagnostics, efforts at infection prevention and clinical care are of great importance for COVID-19 patients. Antibiotic treatment has side effects for the patients (e.g. changes to the microbiome) and the population (selection of resistant pathogens). Guidelines devised in 2021 emphasized the low level of available evidence regarding antibiotic treatment of COVID-19 patients, as well as the importance of appropriate microbiological sampling [29]. A recent study found no difference in patient outcome when antibiotic consumption was reduced by anti-microbial stewardship audits [30].

Our study, as well as others in the past, clearly shows the negative impact of bacterial respiratory or bloodstream superinfections. It therefore seems appropriate to apply the general principles of rational use of antibiotics, although this is undoubtedly challenging in a pandemic.

The currently circulating SARS-CoV-2-variants are associated with milder courses than the ones during this study period and can be expected to be associated with fewer superinfections. Nevertheless, it is important to understand frequency and outcome of bacterial infection in underlying viral infection.

### Limitations of the study

When interpreting our data, some limitations must be taken into account. We performed a retrospective single-center study with a restricted analysis of further patient demographics (e.g. comorbidities and severity of disease) and treatments (antibiotic treatment, immunosuppression), which can have an influence on the mortality. This is primarily due to the fact that only limited structured data were available for evaluation and that treatment regimens were constantly changing, especially at the beginning of the pandemic. We did not stratify our cohort regarding mechanical ventilation or extracorporal-membrane oxygenation (ECMO). Montrucchio et al. [31] recently described among others invasive procedures, ECMO, use of steroids and vasopressor therapy as important risk factors for acquiring a superinfection. We focused on bacterial superinfections without the evaluation of filamentous fungal and viral superinfections due to the limited availability of data on other virus PCRs and structured additional parameters (e.g. fungal serology and PCR) especially needed to verify positive cultures for filamentous fungi.

We assumed that patients without bacterial testing had no superinfection, which probably leads to conservative effect estimates. A notable constraint of our study are the criteria to define the occurrence of superinfections, which was primarily based on the microbiological findings. While the significance of pathogen detection is generally clear in cases of bloodstream infections, it can be difficult to distinguish between colonization and infection in respiratory materials, which may result in an over- or underestimation of superinfections. For this reason, we performed our statistical analysis not only for the totality of infections, but also considered bloodstream infections separately.

## Supplementary Information

Supplementary Material 1

Supplementary Material 2

## Data Availability

The datasets analysed during the current study are not publicly available due to the protection of patient data.
