# Supplementary material for: The burden of nosocomial superinfections in a retrospective cohort study of critically ill COVID-19 patients
Source: BMC Infect Dis. 2025 May 3;25:650. doi: 10.1186/s12879-025-10983-7 (PMC12049767; doi:10.1186/s12879-025-10983-7)
Supplement: Supplementary file 1 — Supplementary Material 1 [file 12879_2025_10983_MOESM1_ESM.pdf]

Supplementary Table 1 Percentages of data shown in Figure 1A

**Pathogen**	**Material**	**Number of pathogen detections**	**Percentage of pathogen detections per material**
Enterobacterales	Blood culture	35	33,65
Nonfermenters	Blood culture	5	4,81
Staphylococcus aureus	Blood culture	14	13,46
Streptococcus pneumoniae	Blood culture	1	0,96
Streptococcus agalactiae	Blood culture	1	0,96
Haemophilus influenzae	Blood culture	0	0
Enterococcus spp.	Blood culture	14	13,46
Coagulase-negative *staphylococci*	Blood culture	22	21,15
Candida spp.	Blood culture	6	5,77
Miscellaneous	Blood culture	6	5,77
Enterobacterales	Respiratory material	126	58,6
Nonfermenters	Respiratory material	30	13,95
Staphylococcus aureus	Respiratory material	43	20
Streptococcus pneumoniae	Respiratory material	7	3,26
Streptococcus agalactiae	Respiratory material	1	0,47
Haemophilus influenzae	Respiratory material	6	2,79
Miscellaneous	Respiratory material	2	0,93

Supplementary Table 2 Percentages of data shown in Supplementary Figure 4

**Pathogen**	**Material**	**Number of patients**	**Percentage of all patients**
Enterobacterales	Blood culture	32	11,94
Nonfermenters	Blood culture	5	1,87
Staphylococcus aureus	Blood culture	14	5,22
Streptococcus pneumoniae	Blood culture	1	0,37
Streptococcus agalactiae	Blood culture	1	0,37
Enterococcus spp.	Blood culture	14	5,22
Coagulase-negative *staphylococci*	Blood culture	20	7,46
Candida spp.	Blood culture	6	2,24
Miscellaneous	Blood culture	6	2,24
Enterobacterales	Respiratory material	96	35,82
Nonfermenters	Respiratory material	29	10,82
Staphylococcus aureus	Respiratory material	43	16,04
Streptococcus pneumoniae	Respiratory material	7	2,61
Streptococcus agalactiae	Respiratory material	1	0,37
Haemophilus influenzae	Respiratory material	6	2,24
Miscellaneous	Respiratory material	2	0,75
