# Supplementary material for: The burden of nosocomial superinfections in a retrospective cohort study of critically ill COVID-19 patients
Source: BMC Infect Dis. 2025 May 3;25:650. doi: 10.1186/s12879-025-10983-7 (PMC12049767; doi:10.1186/s12879-025-10983-7)

Supplementary Figure 1 Inclusion criteria and numbers of patients included in the analysis

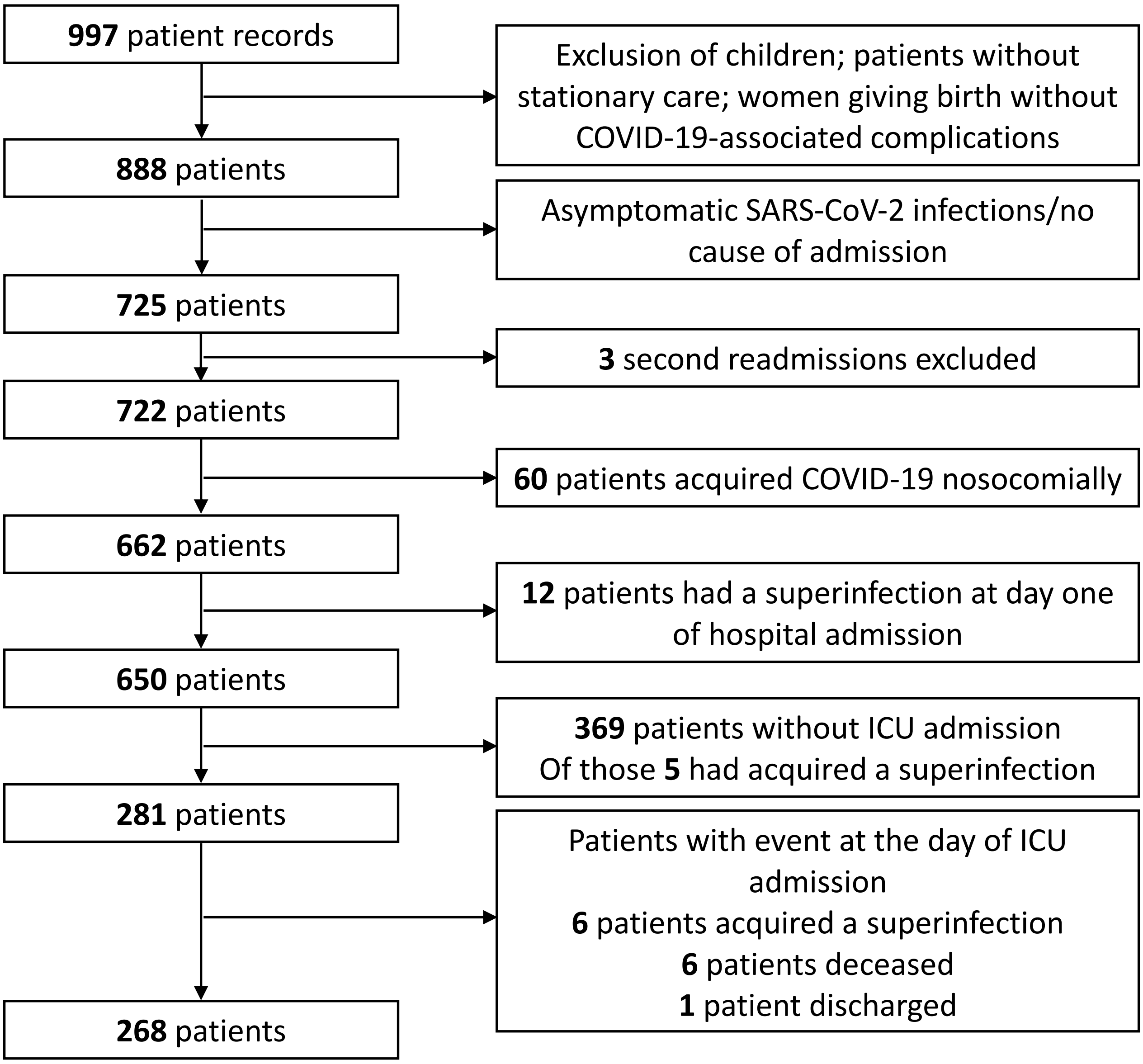

Supplementary Figure 2 Model for the mortality analysis

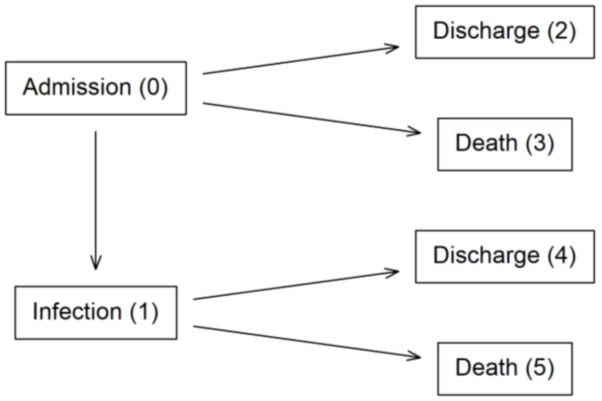

Supplementary Figure 3 Model to calculate the extra length of stay

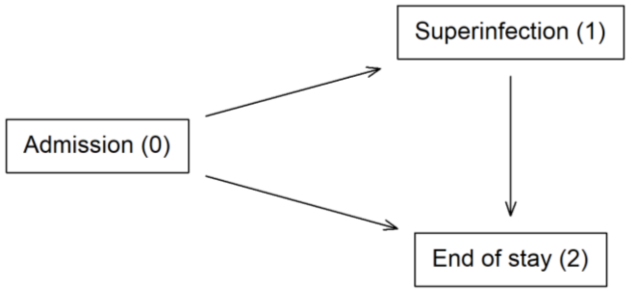

Supplementary Figure 4 Number of patients with at least one of the pathogens detected per material (y-axis) and proportion of all patients (x-axis)

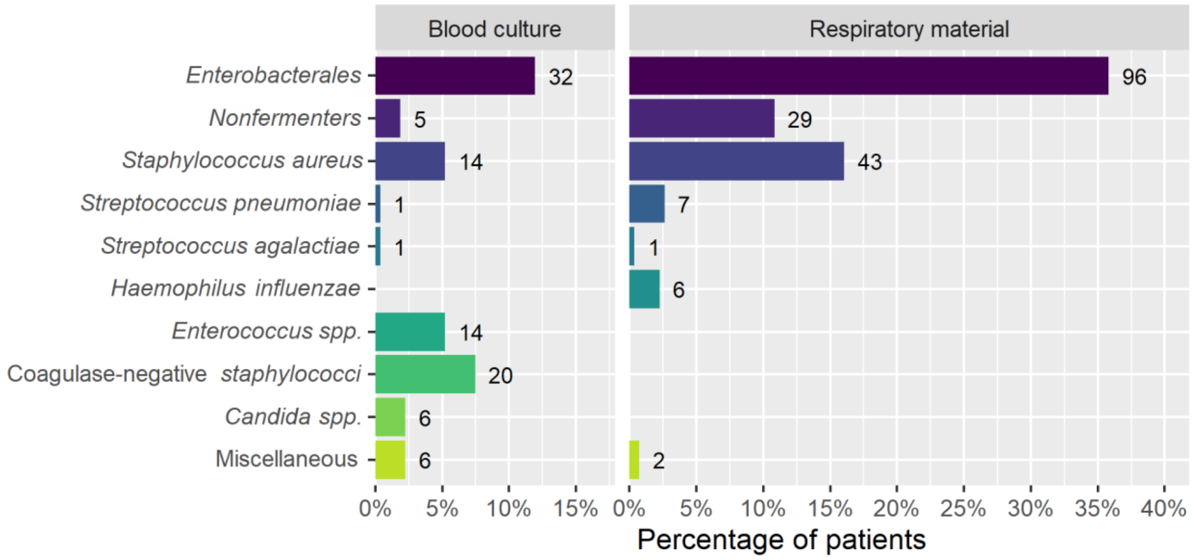

Supplementary Figure 5 Hazard rates of superinfections, discharge without superinfection and death without superinfection in the first 30 days post ICU admission

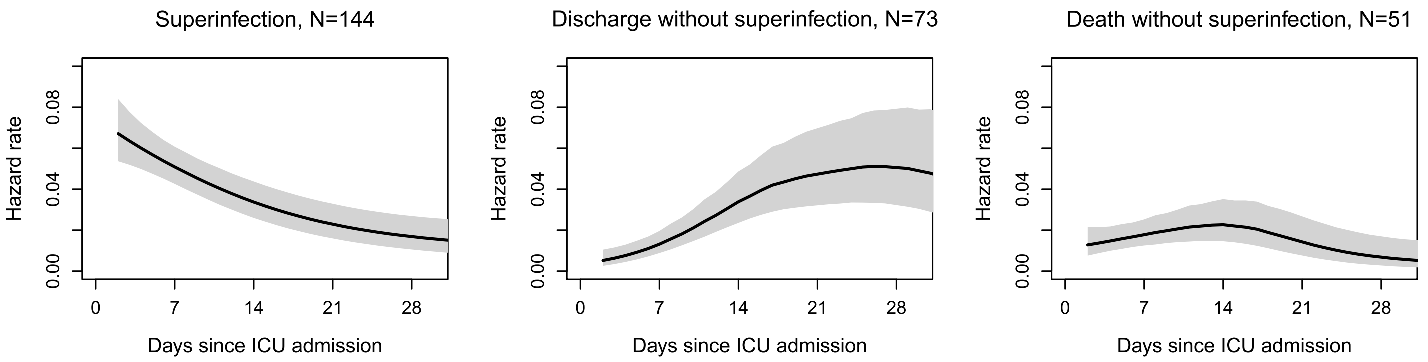

Supplement: Supplementary file 2 — Supplementary Material 2 [file 12879_2025_10983_MOESM2_ESM.pdf]
